# Supplementary material for: Combined Treatment with Acalabrutinib and Rapamycin Inhibits Glioma Stem Cells and Promotes Vascular Normalization by Downregulating BTK/mTOR/VEGF Signaling
Source: Pharmaceuticals (Basel). 2021 Aug 29;14(9):876. doi: 10.3390/ph14090876 (PMC8464793; doi:10.3390/ph14090876)
Supplement: Supplementary file 1 [file pharmaceuticals-14-00876-s001.zip › pharmaceuticals-1319121-supplementary.pdf]

SUPPLEMENTARY INFORMATION

Combined treatment with acalabrutinib and rapamycin inhibits glioma stem cells and promotes vascular normalization by downregulating BTK/mTOR/VEGF signaling

Yu-Kai Su [1,2,3,4,] Oluwaseun Adebayo Bamodu[5,] I-Chang Su[1,2,3,4,] Narpati Wesa Pikatan[5,6,] Iat-Hang Fong[1,2,3,4,] Wei-Hwa Lee[7,] Chi-Tai Yeh [5,8,] Hsiao-Yean Chiu [9*] and Chien-Min Lin [1,2,3,4,*]

1 Graduate Institute of Clinical Medicine, College of Medicine, Taipei Medical University, Taipei City 11031, Taiwan.

2 Department of Neurology, School of Medicine, College of Medicine, Taipei Medical University, Taipei City 11031, Taiwan.

3 Division of Neurosurgery, Department of Surgery, Taipei Medical University-Shuang Ho Hospital, New Taipei City 23561, Taiwan.

4 Taipei Neuroscience Institute, Taipei Medical University, Taipei 11031, Taiwan.

5 Department of Medical Research & Education, Taipei Medical University-Shuang Ho Hospital, New Taipei City 23561, Taiwan.

6 Doctorate Program of Medical and Health Science, Faculty of Medicine, Public Health, and Nursing, Universitas Gadjah Mada, 55281, Yogyakarta, Indonesia.

7 Department of Pathology, Taipei Medical University-Shuang Ho Hospital, New Taipei City, Taiwan.

8 Department of Medical Laboratory Science and Biotechnology, Yuanpei University of Medical Technology, Hsinchu 300, Taiwan.

9 College of Nursing, Taipei Medical University, Taipei, Taiwan.

*Corresponding author

Dr. Chien-Min Lin, Division of Neurosurgery, Department of Surgery, Taipei Medical University-Shuang Ho Hospital, New Taipei City 23561, Taiwan. E-mail: m513092004@tmu.edu.tw

Dr. Hsiao-Yean Chiu, College of Nursing, Taipei Medical University, Taipei, Taiwan. E-mail: hychiu0315@tmu.edu.tw

Supplementary Table S1. Western blot antibodies sheet.

No.	Target	Dilution	MW	Source	
1	SOX2	1:1000	43	ab97959	Abcam
2	4-Oct	1:1000	45	ab200834	Abcam
3	CD133	1:1000	120	ab216323	Abcam
4	NANOG	1:1000	37	ab109250	Abcam
5	GFAP	1:1000	53	ab7260	Abcam
6	NEFL	1:1000	180-220	MA5-14981	Abcam
7	p-BTK	1:1000	77	#5082	Cell Signaling
8	BTK	1:1000	77	#8547	Cell Signaling
9	p-AKT	1:1000	60	#4060	Cell Signaling
10	AKT	1:1000	60	#4685	Cell Signaling
11	p-mTOR	1:1000	289	#5536	Cell Signaling
12	mTOR	1:1000	289	#2983	Cell Signaling
13	p-S6k	1:1000	70	#9204	Cell Signaling
14	S6K	1:1000	70	#2708	Cell Signaling
15	GAPDH	1:1000	42	60004-1-Ig	proteintech
16	ANGPT1	1:1000	14	sc-74528	santa cruz
17	ANGPT2	1:1000	62-70	sc-74403	santa cruz
18	VEGF	1:1000	21	sc-7269	santa cruz
19	Nestin	1:1000	250	MA1-110	Thermo
20	KLF4	1:1000	55	MA5-15672	Thermo

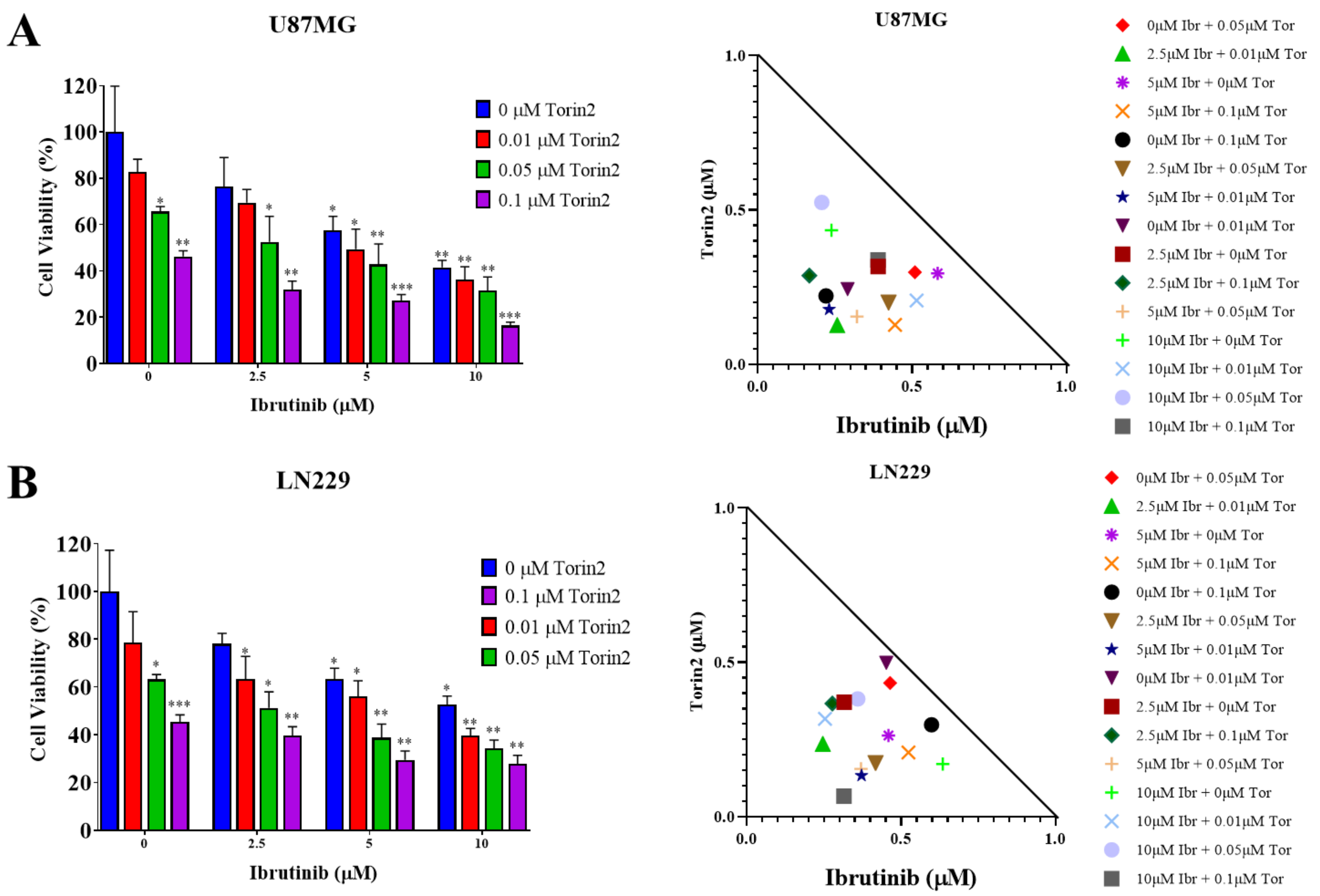

Supplementary Figure S1. Combined Ibrutinib and Torin2 exhibits synergism.

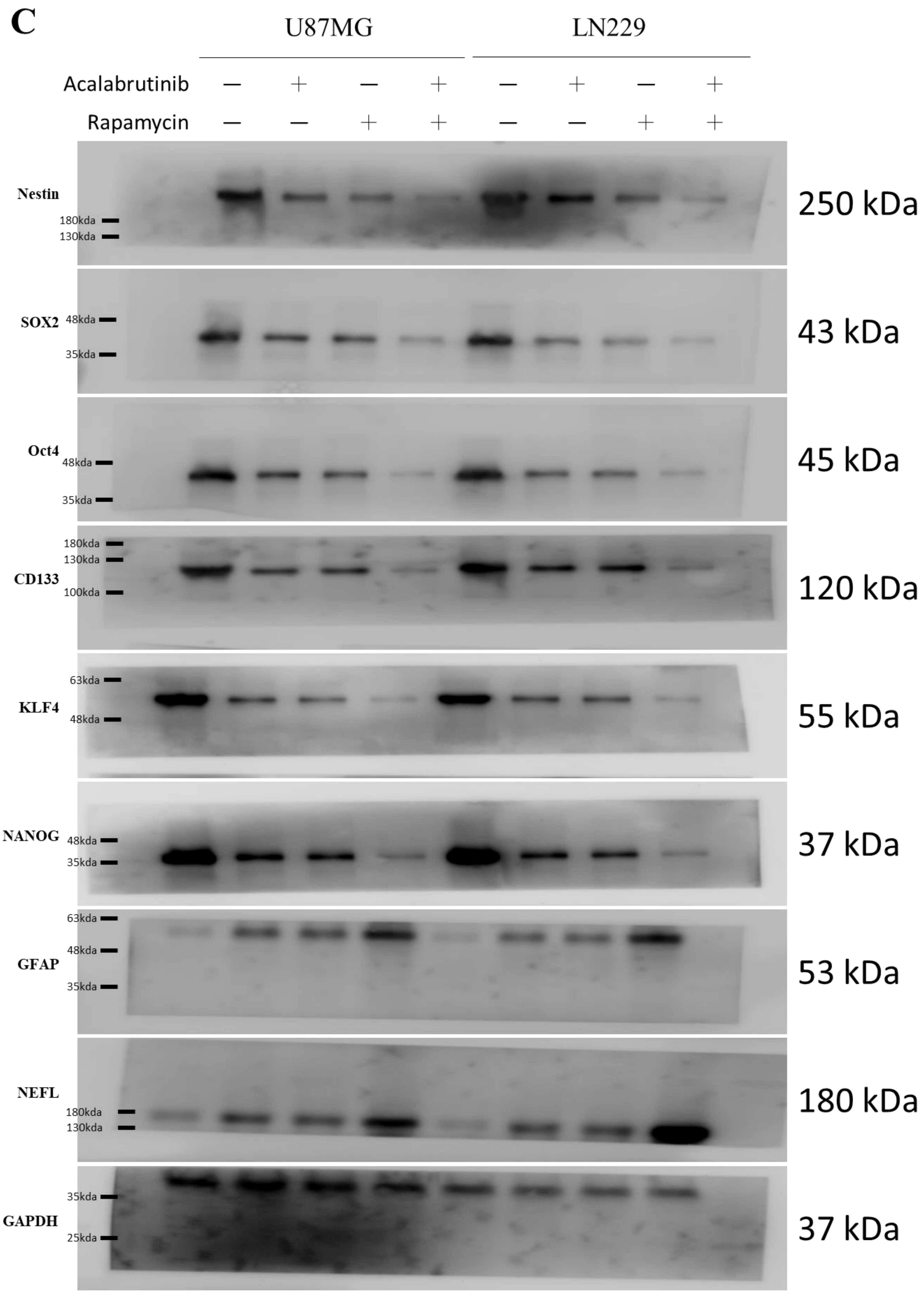

Supplementary Figure S2. Full-size blots of Figure 2C

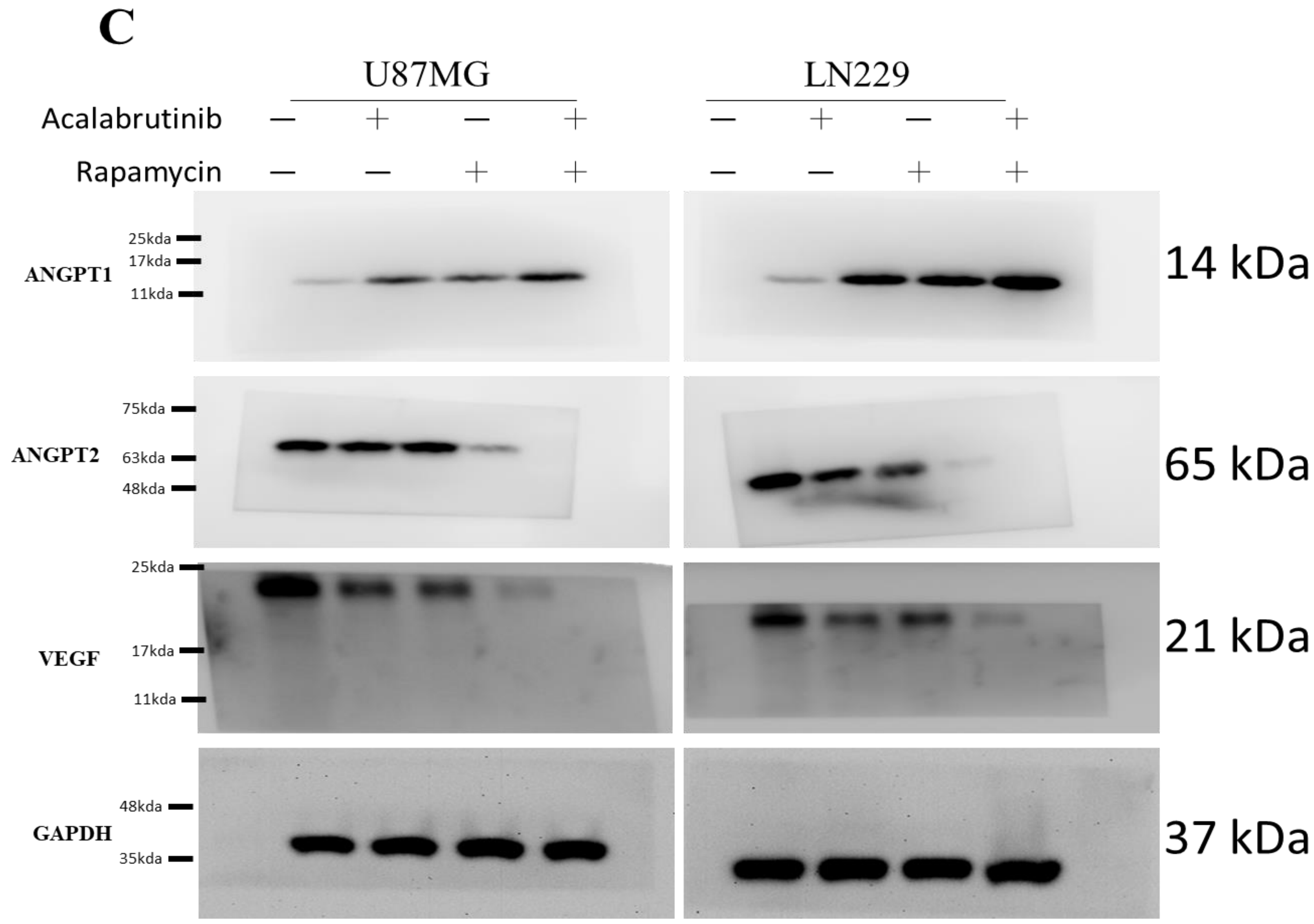

Supplementary Figure S3. Full-size blots of Figure 4C

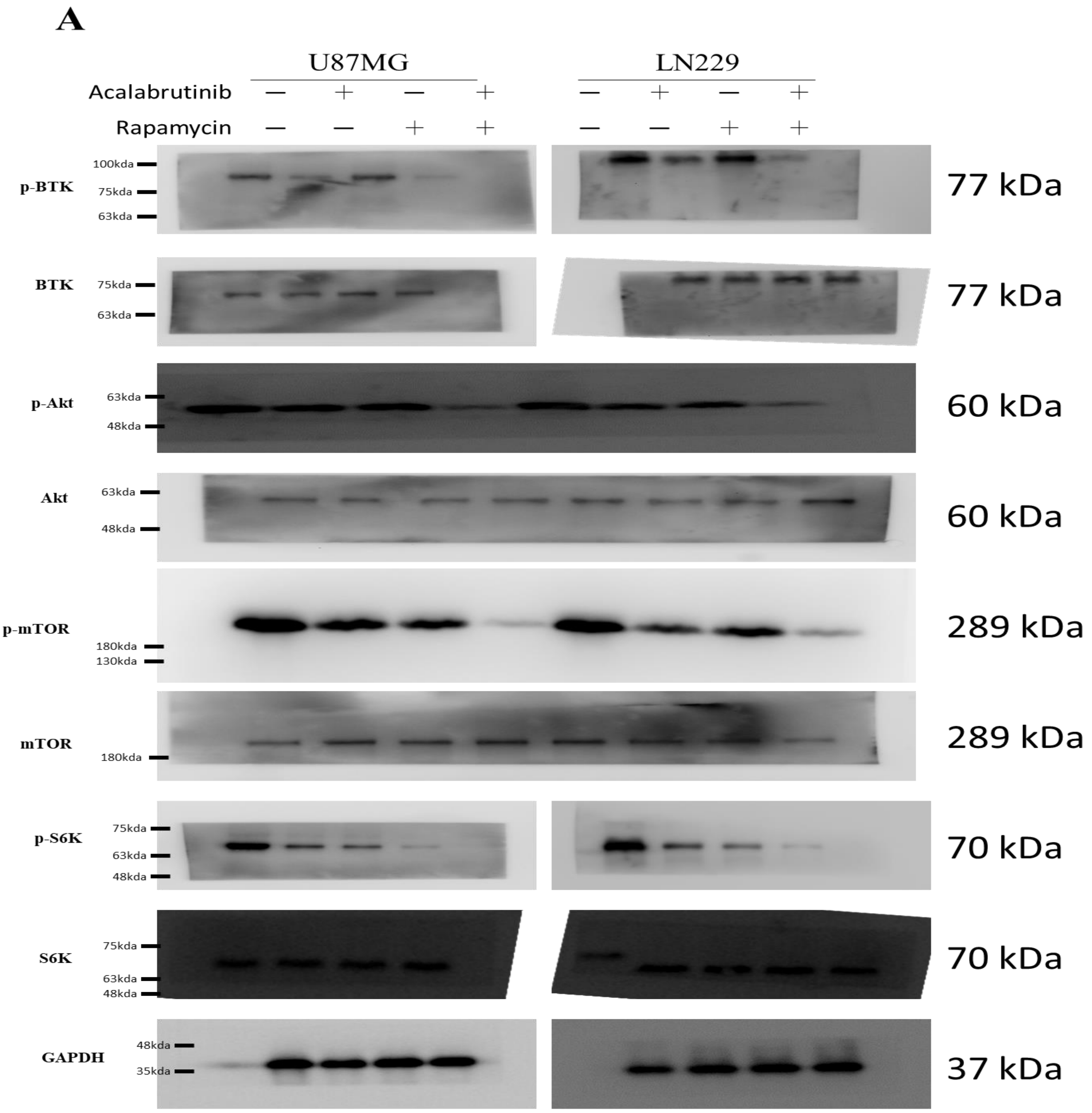

Supplementary Figure S4. Full-size blots of Figure 5A
